# Common-Mode Voltage Reduction in Capacitive Sensing of Biosignal Using Capacitive Grounding and DRL Electrode

**DOI:** 10.3390/s21072568

**Published:** 2021-04-06

**Authors:** Tadeas Bednar, Branko Babusiak, Michal Labuda, Milan Smetana, Stefan Borik

**Affiliations:** Department of Electromagnetic and Biomedical Engineering, University of Zilina, 01026 Zilina, Slovakia; tadeas.bednar@feit.uniza.sk (T.B.); michal.labuda@feit.uniza.sk (M.L.); milan.smetana@feit.uniza.sk (M.S.); stefan.borik@feit.uniza.sk (S.B.)

**Keywords:** capacitive sensing, common-mode noise, noise suppression, grounding, DRL

## Abstract

A capacitive measurement of the biosignals is a very comfortable and unobtrusive way suitable for long-term and wearable monitoring of health conditions. This type of sensing is very susceptible to noise from the surroundings. One of the main noise sources is power-line noise, which acts as a common-mode voltage at the input terminals of the acquisition unit. The origin and methods of noise reduction are described on electric models. Two methods of noise removal are modeled and experimentally verified in the paper. The first method uses a passive capacitive grounding electrode, and the second uses an active capacitive Driven Right Leg (DRL) electrode. The effect of grounding electrode size on noise suppression is experimentally investigated. The increasing electrode area reduces power-line noise: the power of power-line frequency within the measured signal is 70.96 dB, 59.13 dB, and 43.44 dB for a grounding electrode area of 1650 cm^2^, 3300 cm^2^, and 4950 cm^2^, respectively. The capacitive DRL electrode shows better efficiency in common-mode noise rejection than the grounding electrode. When using an electrode area of 1650 cm^2^, the DRL achieved 46.3 dB better attenuation than the grounding electrode at power-line frequency. In contrast to the grounding electrode, the DRL electrode reduces a capacitive measurement system’s financial costs due to the smaller electrode area made of the costly conductive textile.

## 1. Introduction

Bioelectrical signals such as electrocardiography (ECG), electromyography (EMG), and electroencephalography (EEG) are diagnostic methods that are frequently used in medicine to monitor the health and diagnose various diseases. The frequency ranges of these signals are given in Table 1 [1]. Traditional monitoring systems use electrodes (most often Ag/AgCl) placed directly on the measured subject’s skin. An electrically conductive gel in liquid or solid form decreases transient resistance between the electrode and the skin. The electrode-gel combination provides a relatively good conductive contact (resistance < 10 kΩ) and signal transmission between the electrode and the patient’s body. Nevertheless, when wet electrodes are used, the conductive gel dries out during long-term measurements, and the electroconductive gel can cause skin irritation [2]. The use of measurement systems using wet electrodes is not suitable for unobtrusive and long-term sensing because of the measured subject’s discomfort. An alternative to such electrodes is the so-called dry electrodes by which the signal is sensed without direct contact with the patient skin. This kind of electrode can be engaged in chairs or beds. Electrodes with integrated electronics, called active electrodes (AE), are commonly used for this purpose. Such electrodes can be applied directly to the body, or the signal is sensed through the wearable garment. It is advantageous to use electrodes without direct skin contact to create a system for long-term and comfortable monitoring of biosignals.

The AEs with an integrated operational amplifier (OA) are commonly used because they form an impedance barrier between the electrode–skin interface and the signal processing circuits. Sensing using active electrodes leads to certain problems caused by the high impedance of capacitive electrodes in the frequency range of bioelectric signals (Table 1). The electrical schematic of the active electrode, the sensing surface’s size, material, and the dielectric between the electrode and the skin are essential for capacitive electrodes. In Asl et al. [3], the transfer properties of active electrodes were compared using different sensing surface areas (64 cm^2^, 32 cm^2^, 16 cm^2^). The work by Babusiak et al. [4] pointed out the different transfer properties of active electrodes using electrically conductive textile materials made of Shieldex and Elitex fibers. A comparison of three flexible capacitive electrodes was presented by Peng et al. [5 They simultaneously measured the ECG signal using active electrode sensing surfaces made of conductive textile, copper foil tape, and a flexible printed circuit. In Rachim et al. [6], a capacitive electrode’s transmission properties were compared while acquiring an ECG signal through the different dielectric thicknesses. They compared the transmission characteristics for cotton with a thickness of 0.1 mm to 3 mm.

Another issue concerning active electrodes is the common-mode voltage (50 Hz signal for Europe, Africa, Asia, and Australia or 60 Hz for North America and North Latin America) sensed along with the useful signal. In standard sensing, the suppression of this signal is realized by connecting the ground of the system to the patient’s body using a conventional Ag/AgCl electrode or by active rejection using a DRL (Driven Right Leg) circuit [7]. The use of DRL electrode presents the Common-Mode FeedBack (CMFB) method, where the common-mode output voltage of AEs is inverted, gain and fed back to the subject. The use of a conventional electrode connected to the body is ruled out in capacitive non-intrusive monitoring. A capacitive electrode must also replace the standard ground electrode. The capacitively coupled electrodes also have a higher impedance (at a mains frequency of 50 Hz/60 Hz) than conventional ones. One way to reduce the impedance of such an electrode is to increase its area, which increases the cost of the measuring system itself because the conductive fibers are relatively expensive. In work of Lim et al. [8], the impact of various DRL circuit gains on the common-mode voltage was examined. Another option to reduce the common-mode voltage in measured data is the Common-Mode FeedForward (CMFF) technique. This method is based on the compensation of the common-mode signal at both AE inputs before amplification. This kind of method was used, for example, in the works of Xu et al. [9] and [10]. The disadvantage of this method type is a requirement of a specialized circuit design. The next option to suppress common-mode voltage is embedding the additional notch filter inside the acquisition unit [11]. This solution’s disadvantage rests in the signal saturation at the input of active electrodes in case of the high value of common-mode voltage without using the third noise suppression electrode.

For this reason, we decided to create models describing the interference of a capacitively coupled monitoring system with a power-line, which is used to examine the suppression of the common-mode voltage by grounding electrode. In this work, we compare the properties of a system using passive and active suppression of power-line interference. We also created a measurement system to compare the signal sensed by both methods at different sizes of ground electrodes.

## 2. Materials and Methods

**Figure 1:**
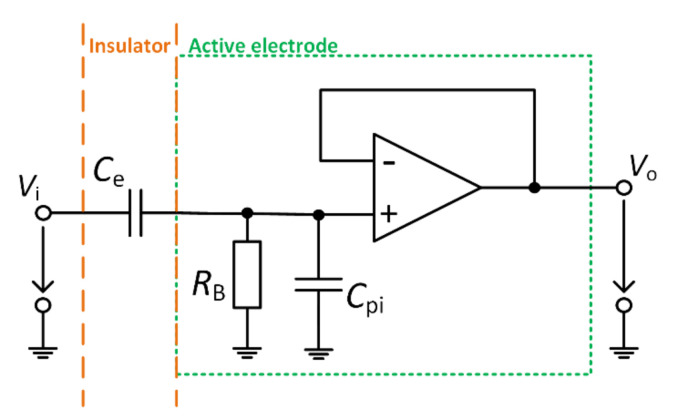
Model of the active electrode.

Figure 1 shows the schematic of the active electrode with OA in the configuration as a voltage buffer, where *C*_e_ represents the capacitance between the sensing area of AE and the subject skin. In some studies, parallel leakage resistance is considered, but it is commonly neglected because of its high value [12]. The real OA has finite input impedance (≈TΩ), unlike the ideal OA, so the bias current flows through OA’s input. When the *C*_e_ capacitance is connected to this input, it is necessary to create an electrical path for bias current to prevent charging of the connected capacitor. The *R*_B_ bias resistor is commonly used for this purpose. The input capacitance of AE also must be considered during the design and construction of AE. The parasitic capacitance is shown as *C*_pi_ in Figure 1. The complex transfer characteristic is stated as

(1) $$G\left(j\omega \right)\;=\;\frac{j\omega C_{e}R_{B}}{1+j\omega R_{B}\left(C_{e}\;+\;C_{\mathrm{pi}}\right)}.$$

As we can see, the *C*_pi_ parasitic capacitance decreases the gain of the active electrode, and the effect of the parasitic capacitance has to be eliminated. A few commonly used methods for parasitic capacitance cancelation exist, for example, neutralization circuit or using of guard layer [13,14,15,16]. When using the assumption of neglected parasitic capacitance, the transfer characteristic can be determined as

(2) $$G\left(j\omega \right)=\;\frac{j\omega C_{e}R_{B}}{1+j\omega R_{B}C_{e}}.$$

The combination of *C*_e_ and *R*_B_ creates the high-pass filter at the beginning of the voltage follower, whose *f*_c_ corner frequency can be calculated according to the Equation:

(3) $$f_{c}\;=\;\frac{1}{2\pi R_{B}C_{e}}.$$

**Figure 2:**
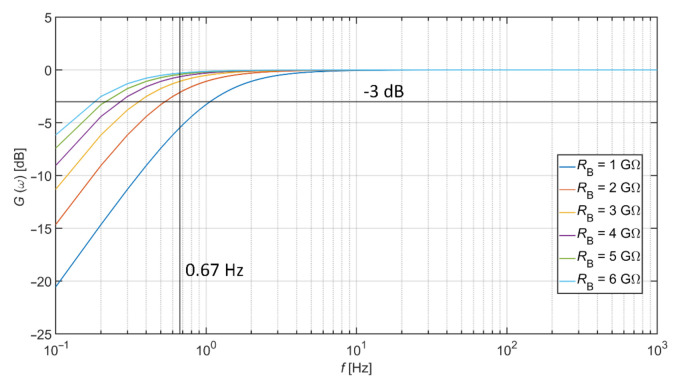
Transfer characteristics according to different values of *R*_B_.

In general, the capacitive electrodes have a sensing area in the range of a few cm^2^, e.g., the work by Bednár et al. [17] presents the sensing area of 16 cm^2^, and the work by Hou et al. [18] uses an area of 12.56 cm^2^. Assuming such size of the sensing surface (assumption with clothes with thickness less than a few mm), the *C*_e_ capacitance between the skin’s surface and the electrode reaches a value range from 10 pF to a few hundreds of pF. This fact implies that the high-value bias resistor (GΩ) must be used to acquire bioelectrical signals in the required frequency bandwidth. The *R*_B_ bias resistor dependent transfer characteristic, with the assumption of *C*_e_ = 150 pF (also used in work of Babusiak et al. [4]), is shown in Figure 2. This figure shows that the usage of capacitively coupled electrodes is not appropriate for the measurement of diagnostic ECG. However, they can monitor ambulatory ECG [19] or surface EMG [20]. The figure also shows that the *R*_B_ bias resistor value of 2 GΩ is sufficient for monitoring ambulatory ECG while using the capacitive coupling of 150 pF.

The active electrode with integrated circuit AD8642 (Analog Devices, Inc., Norwood, MA, USA) with two OAs was designed for our measurements. Both OAs were configurated as voltage buffers. The first one was used as an impedance barrier (Figure 1), and the second one creates a guard layer to reduce the effect of parasitic input capacitance. The designed AE was constructed as 3-layer PCB (Printed Circuit Board). The bottom layer represents the sensing layer, the middle layer represents the guard, and the top layer includes integrated OAs with a bias resistor and ground plane around components.

**Figure 3:**
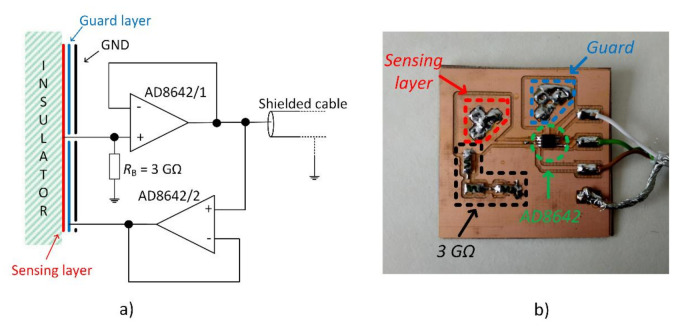
Schematic (**a**) and design (**b**) of the constructed active electrode. Reprinted with permission from ref. [16]. Copyright 2019 Elsevier B.V.

The same electrode was presented and analyzed in detail by Bednar et al. [16]. Figure 3a represents the schematic and layers disposition of AE Figure 3b shows the physical construction of AE with a sensing area of 16 cm^2^. The impact of the active electrode guard layer in capacitively coupled measurements was already presented by Bednar et al. [16]. In this work, the improvement of the AE’s transfer characteristic was confirmed due to the use of the guard layer. Moreover, it was proved that the guard layer increases the total CMRR (Common Mode Rejection Ratio) of the acquisition system.

**Figure 4:**
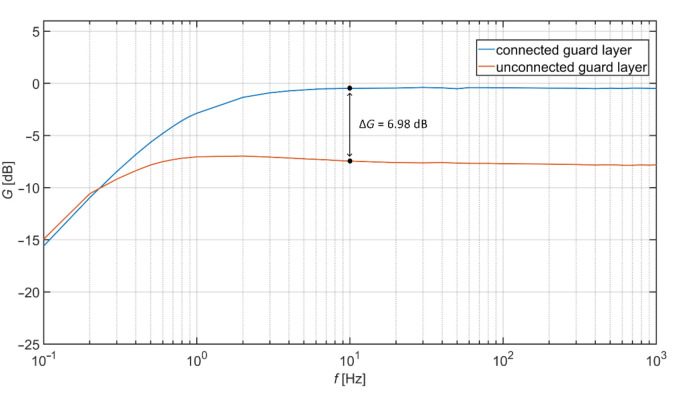
Measured transfer characteristics.

Comparison of transfer characteristics with connected and unconnected guard layer is shown in Figure 4. The cotton with a thickness of 0.45 mm is used as a dielectric material, and the pressure of 1.23 kPa was applied to the electrode. The comparison shows that the involvement of the guard layer improves the transfer properties of AE. For example, the gain difference of the unguarded and guarded electrodes is 6.98 dB at 10 Hz.

**Figure 5:**
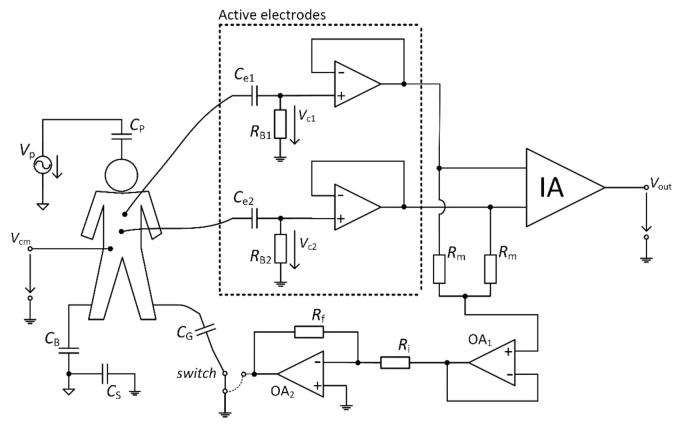
Electrical model depicting the power-line interference.

The electrical model of the acquisition system for capacitive measurement is shown in Figure 5. The model is based on similar models presented in [11,21,22]. The parasitic capacitances between the power-line, measured subject, and the device are also considered. The *V*_P_ represents the power-line source, *C*_P_ capacitance between the power-line and the measured subject, *C*_B_ capacitance between the subject and the power-line ground, and *C*_s_ capacitance between the ground of the acquisition system and the power-line ground. Values of these capacitances are listed in Table 2 [11,21,22]. These values are indicative, and they depend on the methodology of measurement, environment, and parameters of the measuring device.

In general, the Ag/AgCl electrodes are used for patient grounding to reduce common-mode noise. The grounding can be realized in two ways: 1. direct connection of the electrode to the ground of the acquisition system; 2. connection of the electrode to specialized DRL (Driven Right Leg) circuit to suppress the common-mode noise actively [7]. This connection decreases the value of *V*_cm_ common-mode voltage measured together with biosignal. The connection of such electrodes is not possible in the case of a capacitively coupled system. The contact grounding electrode has to be replaced by the capacitive noise suppression electrode (NSE). This capacitive electrode is also connected to the ground of the system [23] or to the output of the DRL circuit [24] in the same manner as the standard wet Ag/AgCl electrode. These two options are selectable by a switch in Figure 5. The capacitance between the body and NSE is shown as *C*_G_ in the schematic.

**Figure 6:**
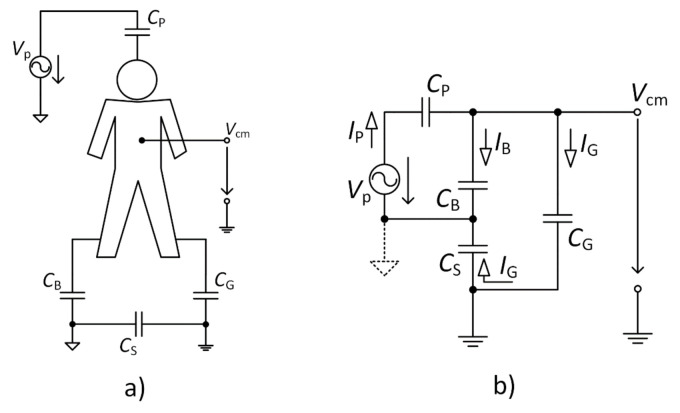
Schematic circuit (**a**) and equivalent circuit for a common-mode voltage (**b**).

Figure 6 shows the simplified model for suppressing *V*_cm_ when the NSE is connected directly to the ground of the acquisition system. Figure 6a shows the interference model, and Figure 6b represents the schematic of this model. In this analysis, we are not dealing with the parameters of AEs because of their high impedance (when we assume the bias resistor in the range of gigaohms).

Based on the model, we can define the ratio between the *V*_cm_ common-mode voltage and *V*_P_ power-line source as

(4) $$\frac{V_{\mathrm{cm}}}{V_{p}}\;=\;\frac{Z_{B}Z_{G}}{Z_{P}Z_{B}\;+\;Z_{P}Z_{S\;}+\;Z_{P}Z_{G\;}+\;Z_{S}Z_{B\;}+\;Z_{G}Z_{B}}\;,$$

where Z_B_, Z_P_, Z_S,_ and Z_G_ are impedances of C_B_, C_P_, C_S,_ and C_G_, respectively. According to that fact, we can derive equation as

(5) $$\frac{V_{\mathrm{cm}}}{V_{p}}\;=\;\frac{C_{P}C_{S}}{C_{S}C_{G}\;+\;C_{G}C_{B\;}+\;C_{B}C_{S\;}+\;C_{P}C_{E\;}+\;C_{S}C_{P}}.$$

Using Equation (5), we can state that with the increasing value of *C*_G_ the suppression of the *V*_cm_ also increases. In the case of infinity *C*_G_ capacitance (*C*_G_ →∞), the *V*_cm_ equals zero.

**Figure 7:**
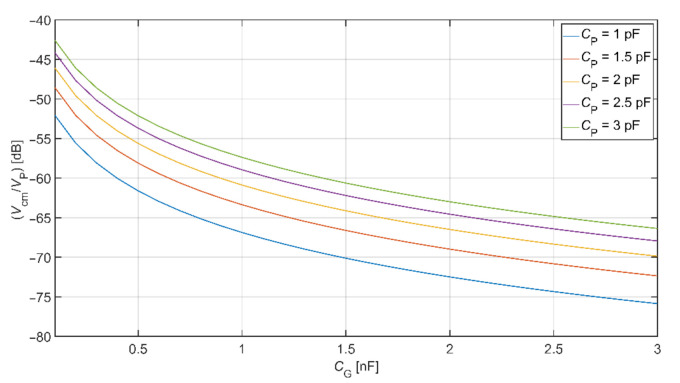
Common-mode voltage rejection for a system with noise suppression electrode (NSE) electrode connected to the system ground.

The grounding electrode size is usually more extensive than the active electrode size. We assume the capacitances are in the range of hundreds pF to a few nF. As mentioned before, the *C*_P_ capacitance changes with the measured subject’s position within the measurement space. This capacitance is changing with the count of other devices connected to the same power network supplied by *V*_P_ and their distances from the measured subject [25]. The value of *C*_P_ is lower while using a lower number of supplied devices. The values of *C*_B_ and *C*_S_ are considered constant in our analysis (we used values listed in Table 2). Figure 7 shows the graphical representation of Equation (5) with the usage of before mentioned values. We can see that the decrease of *V*_cm_ depends on the size of the capacitive grounding electrode and the capacitance between the subject and V_P_ source.

**Figure 8:**
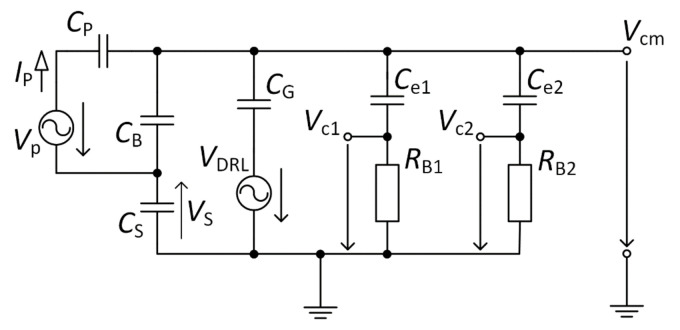
Equivalent schematic of an interference model for the system using a Driven Right Leg (DRL) circuit.

The interference model with a connected DRL circuit is shown in Figure 8. According to Figure 5 (case with a connected switch to the output of OA2), this schematic was created. Figure 6 presented a model where NSE is connected to the ground of the acquisition system, and parameters (*R*_B1_, *R*_B2_, *C*_e1_, *C*_e2_) of AEs were neglected. We have to consider parameters of AEs under the analysis of the *V*_cm_ suppression using the DRL circuit because the DRL circuit inverts and amplifies the mean of AEs output signals. The total suppression of *V*_cm_ depends on the voltages acquired by active electrodes (shown in Figure 8 as *V*_c1_ and *V*_c2_) and on DRL circuit gain. Using the schematic presented in Figure 5, we can define the *G*_DRL_ gain of the DRL circuit by

(6) $$G_{\mathrm{DRL}}=\;-\frac{R_{f}}{R_{i}},$$

and the *V*_DRL_ shown in Figure 8 is defined by

(7) $$V_{\mathrm{DRL}}=G_{\mathrm{DRL}}\left(\frac{V_{c1}\;+\;V_{c2}}{2}\right)=\;-\frac{R_{f}}{R_{i}}\left(\frac{V_{c1\;}+\;V_{c2}}{2}\right).$$

It is possible to define Equations (8)–(11) using Kirchhoff’s laws, based on the model shown in Figure 8. A similar derivation was presented in [22].

(8) $$j\omega C_{P}\left(V_{P}-V_{\mathrm{cm}}-V_{S}\right)\;=\;j\omega C_{B}\left(V_{\mathrm{cm}}+\;V_{S}\right)+j\omega C_{S}V_{S},$$

(9) $$j\omega C_{S}V_{S}=\;j\omega C_{G}\left[V_{\mathrm{cm}}-\;\left(-\frac{R_{f}}{R_{i}}\left(\frac{V_{c1}\;+\;V_{c2}}{2}\right)\right)\right]\;+\;j\omega C_{e1}\left(V_{\mathrm{cm}}\;-\;V_{c1}\right)\;+\;j\omega C_{e2}\left(V_{\mathrm{cm}}-\;V_{c2}\right),$$

(10) $$j\omega C_{e1}\left(V_{\mathrm{cm}}-V_{c1}\right)\;=\;\frac{V_{c1}}{R_{B1}},$$

(11) $$j\omega C_{e2}\left(V_{\mathrm{cm}}-V_{c2}\right)\;=\;\frac{V_{c2}}{R_{B2}}.$$

**Figure 9:**
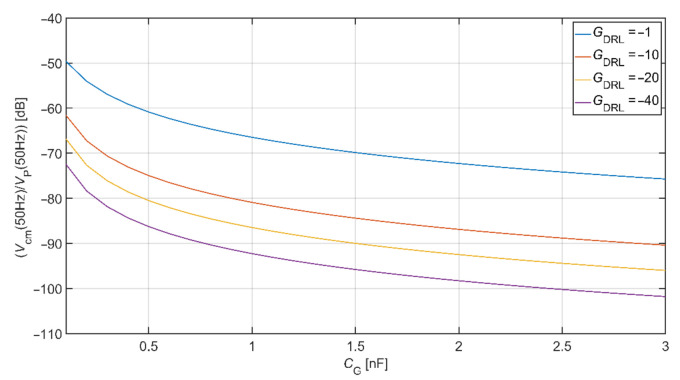
Common-mode voltage rejection for the system using a DRL circuit.

The MATLAB language script was created to evaluate the *V*_cm_ value depending on *C*_G_ capacitance and *G*_DRL_ gain. Equations (8)–(11) were implemented in this script. The values of *C*_B_, *C*_P,_ and *C*_S_ were set up according to Table 2, *C*_e1_ = *C*_e2_ = 150 pF and *R*_B1_ = *R*_B2_ = 3 GΩ. The result of this analysis is shown in Figure 9. It is seen that the value of *V*_cm_ is attenuating with the increasing value of *C*_G_ and *G*_DRL_.

**Figure 10:**
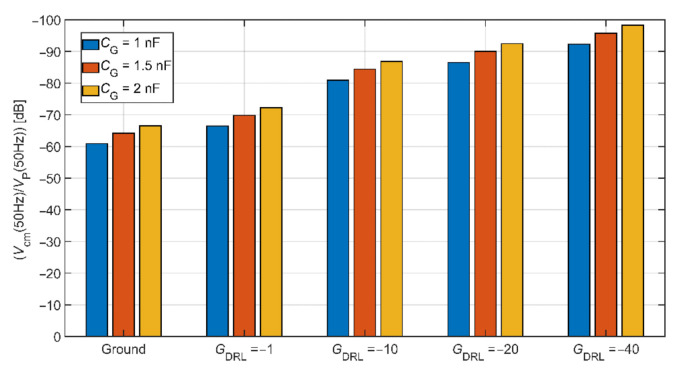
The comparison of common-mode rejection depending on different DRL gain.

The comparison of common-mode rejection depending on different DRL gain is shown in Figure 10. This graph is made for common-mode voltage at a frequency of 50 Hz. Values of coupled capacitances between the subject and the power-line were set up according to Table 2. In the analysis, we assume the value of *C*_e1_ = *C*_e2_ = 150 pF and *R*_B1_ = *R*_B2_ = 3 GΩ. This comparison shows that using the DRL circuit and its increasing gain also increases the system’s common-mode voltage rejection. For example, if *C*_G_ = 1 nF and *G*_DRL_ = −40, the suppression of common-mode voltage is −60.88 dB and −92.28 dB for electrodes connected to system ground and DRL circuit, respectively.

**Figure 11:**
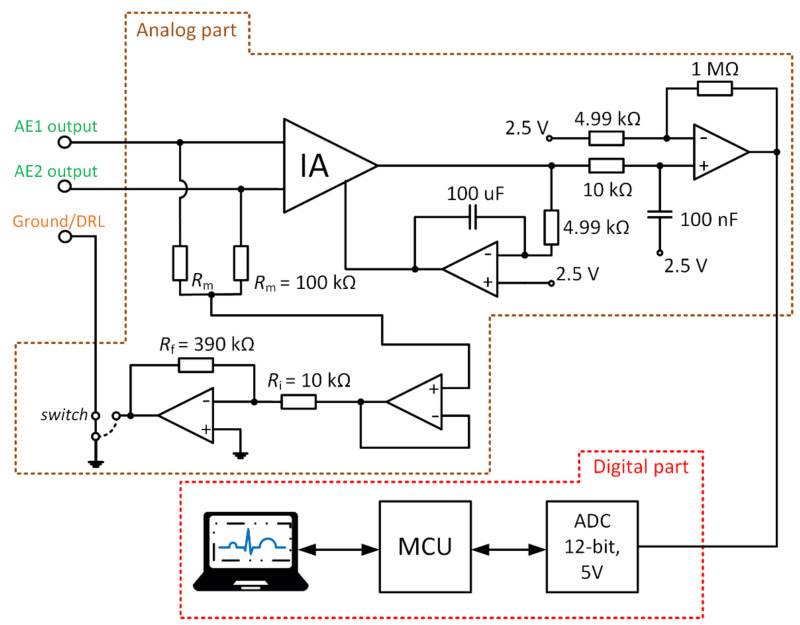
Block scheme of developed electrocardiography (ECG) system.

Figure 11 represents the scheme of the designed acquisition system for measurement of capacitive ECG. The digital and analog parts can be identified in the schematic. The analog part uses an instrumentation amplifier (IA.) AD8421 (Analog Devices, Norfolk County, MA, USA) at the input and 4 OAs AD8642 (Analog Devices, Norfolk County, MA, USA). The analog part is supplied by ±12 V bipolar voltage source. The IA deals with acquiring the signals that come from AEs. The OAs provide additional signal gain, filtration, and take part in the DRL circuit. The developed device allows choosing between the GND and the DRL for the NSE connection. The *G*_DRL_ was set to the value of –39. The digital part provides data digitalization and transmission to the PC. The 12–bit ADC (Analog to Digital Converter) LTC1296 (Linear Technology, Linear Technology, USA) with a 5 V reference was used for digitalization. The ATmega328P (Atmel, San Jose, CA, USA) was used as a control MCU. The gain of IA is 1, and the gain of the successive operational amplifier is set to a value of 201.4. The sampling frequency was set to a value of 500 Hz.

**Figure 12:**
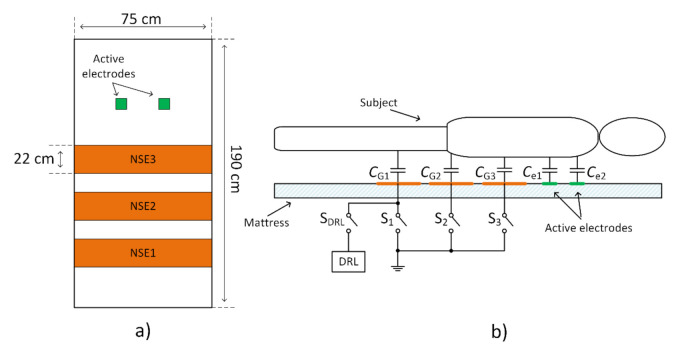
Placement of electrodes on the bedsheet (**a**), and a model of coupling capacitances between the subject and electrodes (**b**).

Three NSEs with the size 22 cm × 75 cm and two AEs were placed onto the mattress to experimentally examine the dependency between the system’s common-mode rejection and NSE size. The placement of electrodes is shown in Figure 12a. Similar electrode placement was used in previous papers dealing with monitoring of ECG during sleep [24,26,27,28]. NSEs are made from electroconductive fabric. This fabric is made from a fiber with a core from polyamide and a sheath from pure silver. The same fabric was used by Babušiak et al. [28]. The coupling capacitances between the subject body and electrodes are depicted in Figure 12b. These capacitances depend on the position of examined subject and clothes (dielectric) properties. The total capacitance between the subject and NSEs can be defined as the sum of capacitances connected in parallel. In the case of closed switches S_1_, S_2_, and S_3,_ the total capacitance is calculated as

(12) $$C_{G}=C_{G1}+\;C_{G2}+C_{G3}.$$

**Figure 13:**
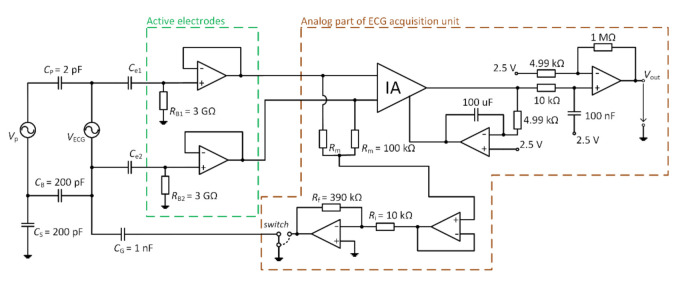
Schematic of the simulation model.

**Figure 14:**
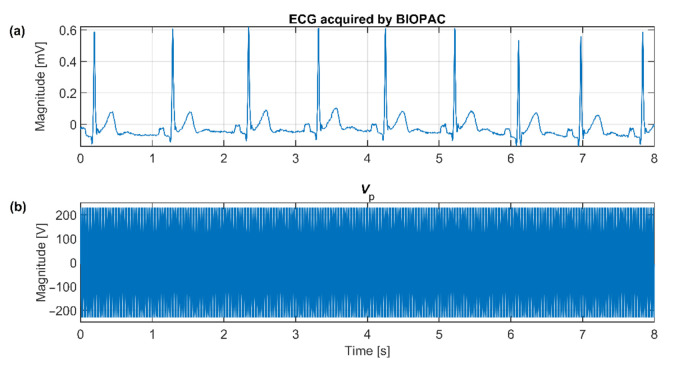
The ECG signal waveform (**a**) and the power-line voltage waveform (**b**) used in the simulation.

The upper presented theory and the as-designed capacitive system are verified by simulation in TINA TI (Texas Instruments, Inc., Dallas, TX, USA). The equivalent model is shown in Figure 13. In the simulation, the macro models of used components are provided by the producer. All parameters are set up according to the capacitive ECG system showed in Figure 11. Capacitances listed in Table 2 are included in our simulation. The *V*_P_ is configured as a sine-wave voltage generator with a frequency of 50 Hz and an amplitude of 230 V (power supply in the EU). The *V*_P_ is used as a source of common-mode voltage. The value of *C*G capacitance is set up to the value of 1 nF. The *V*_ECG_ source generates an ECG signal acquired by bipolar measurement of ECG from the subject’s back using standard disposable electrodes. The placement of disposable electrodes corresponds to the position of the electrodes used in our bed (Figure 12a). The Biopac MP36 acquisition unit (BIOPAC Systems, Goleta, CA, USA) was used for acquiring ECG samples for the *V*_ECG_ source. It was set up hardware band-pass filter with corner frequencies 0.05 Hz and 150 Hz, and sampling frequency was set up to the value of 500 Hz. The waveforms of *V*_ECG_ and *V*_P_ are shown in Figure 14.

## 3. Results and Discussion

**Figure 15:**
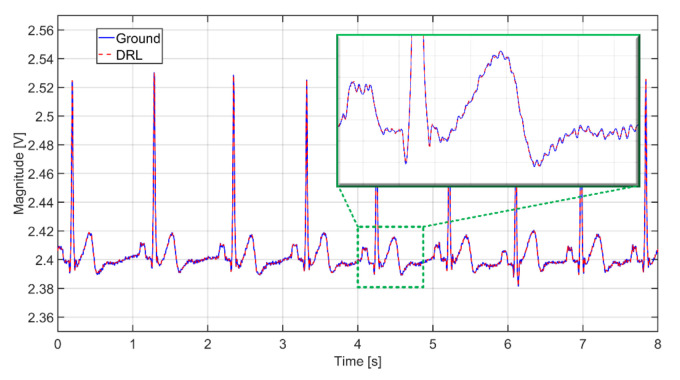
The output signals from simulation while using the same coupling capacitances (*C*_e1_ = *C*_e2_ = 150 pF) at the input in two scenarios: The NSE connected to the ground (solid blue), and NSE connected to the output of DRL (dashed red).

The verification of the presented theory was done by a simulation on the model presented in Figure 13. The first series of the simulation uses the same values of AEs coupling capacitances (*C*_e1_ = *C*_e2_ = 150 pF). The *V*_out_ output signals of NSE connected to the ground and the DRL output are shown in Figure 15. As seen from this figure, the common-mode voltage is effectively suppressed in both cases when the coupling capacitances are identical.

**Figure 16:**
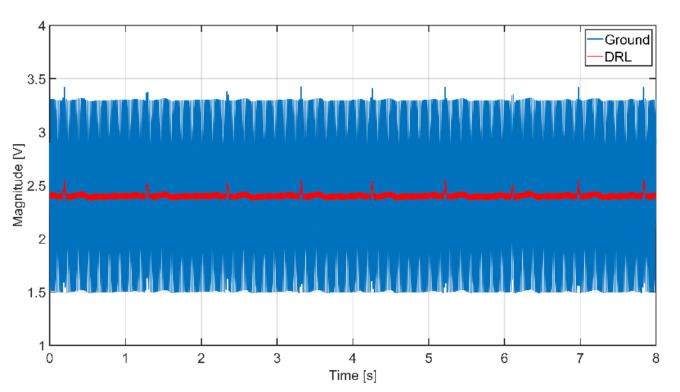
The output signals from simulation while using different coupling capacitances (*C*_e1_ = 150 pF, *C*_e2_ = 75 pF) at the input in two scenarios: The NSE connected to the ground (solid blue), and NSE connected to the output of DRL (solid red).

In practice, it is almost impossible to ensure the same values of input coupling capacitances. This fact is caused by local differences in the thickness and composition of dielectric material (clothes) and the electrode’s pressure. These differences lead to the different gain of each electrode [16]. According to Peng et al. [29], the differences between the input AE capacitances can be doubled during the measurement. We adjusted the simulation parameters to fulfill mentioned assumption: the *C*_e1_ and *C*_e2_ are set up to 150 pF and 75 pF, respectively. A different gain of the AEs leads to an unequal value of the common-mode voltage at the input of IA (see Figure 13). The successive operational amplifier further amplifies the common-mode voltage from the IA output. The simulated output signals corresponding to scenarios with different switch positions are shown in Figure 16. We can see that the unequal gain of AEs caused a relatively high value of the common-mode voltage (50 Hz) in the acquired signal. We can also see that the common-mode voltage is higher when the NSE is connected to the ground than connected to DRL output. The higher *V*_cm_ value or higher difference of input coupling capacitances can cause the saturation of the analog amplifiers or saturation of ADC input. In practice, it is necessary to prevent this kind of problem by sufficient rejection of the *V*_cm_ voltage or balancing input capacitances [30].

A series of experiments were performed to support the results from the simulation. The ECG system in Figure 11 and the electrode placement in Figure 12 are used in the experiments. The measured subject is lying on the mattress, while the bipolar capacitive ECG is measured from the back. A cotton T-shirt and a cotton tracksuit were used as upper-body and lower-body clothes, respectively.

**Figure 17:**
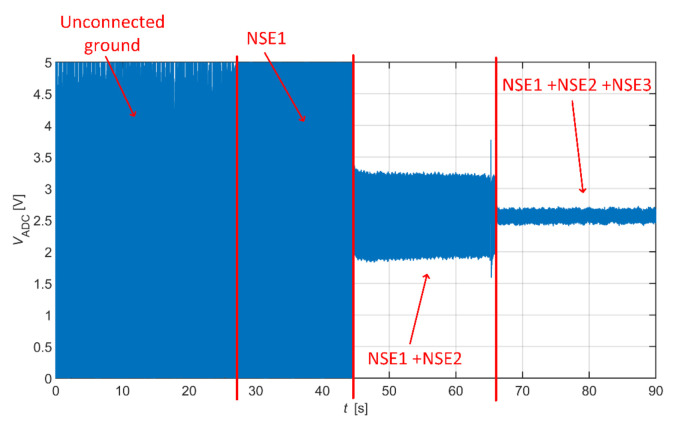
Capacitive ECG with gradually connected grounding electrodes.

The effectivity of the NSE connected to system ground in common-mode rejection was examined at first. All three NSEs are not connected to the system ground at the beginning of the measurement (beginning of signal in Figure 17), and then they are gradually connected to the ground (rest of Figure 17). The ADC input saturation is observed in situations with unconnected NSEs and with one NSE connected due to a high level of common-mode noise. The saturation is not visible when at least two electrodes are connected. The common-mode rejection increases with an increasing number of connected NSEs.

**Figure 18:**
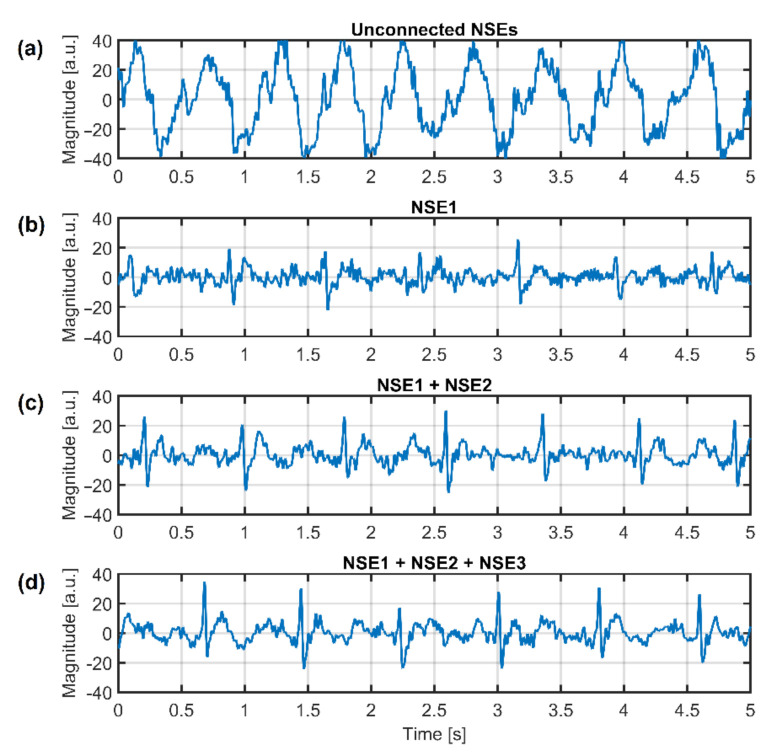
Filtered ECG data with gradually connected grounding electrodes—no grounding electrode (**a**), one grounding electrode (**b**), two grounding electrodes (**c**), and three grounding electrodes (**d**).

As we already stated, the use of AEs is sufficient to measure ambulatory ECG in the frequency range from 0.67 Hz to 40 Hz, so the resulting signal from Figure 17 is filtered by this digital band-pass filter. The filter attenuation in bandstop is 80 dB. The filtered data are shown in Figure 18. The filtered data points to the fact that the system without grounding is saturated, and it is impossible to acquire ECG data. The ECG signal acquired by the system using one NSE (only NSE1) is recognizable, but its quality is shallow. The quality of measured ECG is better when using two and three NSEs.

An extensive area of the grounding electrode made of conductive textile causes a high cost of the electrode as well as the whole system. The DRL circuit is an alternative for common-mode noise reduction in conventional and capacitive systems. In the presented theory, the DRL is more effective in common-mode noise suppression than grounding, as shown in Figure 10.

**Figure 19:**
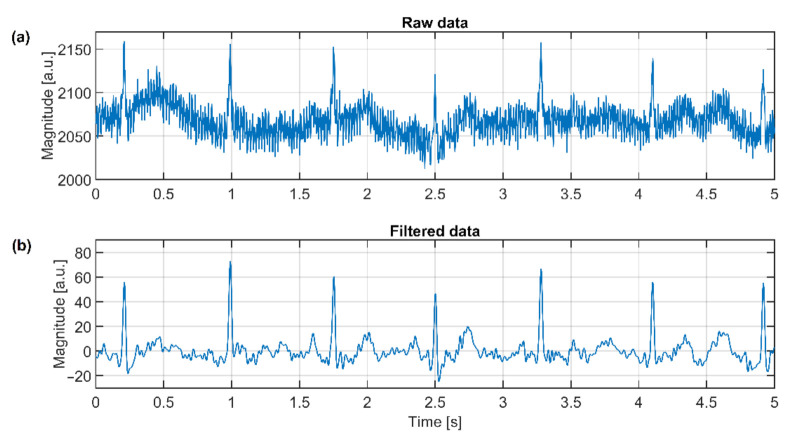
Raw ECG signal (**a**) and filtered signal (**b**) acquired by the capacitive system using the DRL electrode.

The verification of the DRL effectivity is done by measurement using the same system in Figure 11. The NSE1 is connected to the output of the DRL circuit (see Figure 12). The NSE2 and NSE3 are unconnected. The gain of the DRL circuit is –39. The raw and filtered ECG data are shown in Figure 19. The R peaks are fully recognizable in the ECG signal even if only one NSE is used.

**Figure 20:**
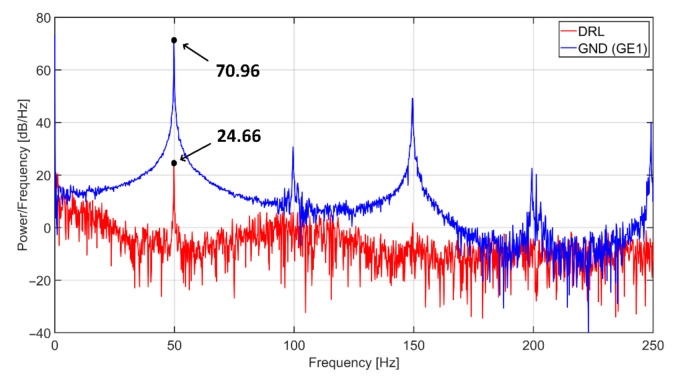
Power spectral densities of capacitive ECG signal using grounding (**blue**) and DRL (**red**) electrode.

The comparison of power spectral densities of the signal acquired by the capacitive system using passive ground and DRL with the same NSE (position of NSE1 electrode) is shown in Figure 20. The difference between the power at a frequency of 50 Hz reaches 46.3 dB. The comparison also shows that the noise is repeated at the multiples of power-line frequency when using a grounding electrode.

The following table summarizes the power of power-line noise (50 Hz) in the resulting ECG signal when using different noise suppression techniques and the number of NSE. The names of NSE are adapted from Figure 12.

## 4. Conclusions

The article was focused on analyzing a system for capacitive measurement of biosignals. In this work, we compared two methods for suppression of common-mode voltage in acquired data by using grounding and DRL electrode. We created the equivalent models describing the power-line’s interference with the subject and the acquisition system (Figure 5, Figure 6, Figure 7 and Figure 8). The models were analyzed in detail by simulations. The models were verified by measurements of capacitive ECG with a living subject lying on the mattress. Although some authors already presented the effect of grounding and DRL circuit on noise suppression, we provide a relevant comparison of these two methods in one article. The suppression methods are experimentally evaluated using one acquisition system, keeping the same condition of measurement. We found out that with the increasing size of the grounding electrode, the reduction of common-mode voltage also increases (Figure 17) according to theoretical assumption. Unfortunately, the large grounding electrode increases the manufacturing cost of the capacitive system due to expensive conductive fabrics. A DRL electrode is a powerful alternative to a grounding electrode. It provides better common-mode rejection (Table 3) while using a smaller electrode area.

## Figures and Tables

**Table 1:** Frequency ranges of biosignals. Reprinted with permission from ref. [1]. Copyright 2017 Elsevier B.V.

Biosignal	Frequency Bandwidth [Hz]
ECG—ambulatory monitoring	0.67–40
ECG—diagnostic purposes	0.05–150
EEG	0.1–100
EMG	10–2000

**Table 2:** Values of coupling capacitances in the power-line and measured subject environment.

Variable	Value [pF]
*C_p_*	2
*C_B_*	200
*C*_S_	200

**Table 3:** The common-mode power of different noise suppression methods.

Noise Suppression Method	Electrodes in Use	Active Electrode Area [cm^2^]	Power of Noise at 50 Hz [dB]
Grounding	NSE1	1650	70.96
	NSE1 + NSE2	3300	59.13
	NSE1 + NSE2 + NSE3	4950	43.44
DRL	NSE1	1650	24.66

## Data Availability

The data presented in this study are available on request from the corresponding author.
